# Insights Into the Evolution of the *prdm1*/Blimp1 Gene Family in Teleost Fish

**DOI:** 10.3389/fimmu.2020.596975

**Published:** 2020-10-30

**Authors:** Pedro Perdiguero, Maria C. Goméz-Esparza, Diana Martín, Steve Bird, Irene Soleto, Esther Morel, Patricia Díaz-Rosales, Carolina Tafalla

**Affiliations:** ^1^ Animal Health Research Center (CISA-INIA), Madrid, Spain; ^2^ Biomedical Unit, School of Science, University of Waikato, Hamilton, New Zealand

**Keywords:** B lymphocyte-induced maturation protein (Blimp1), homolog of Blimp1 in T cells (Hobit), teleost fish, B cells, plasmablasts

## Abstract

In mammals, Blimp1 (B lymphocyte-induced maturation protein 1) encoded by the *prdm1* gene and its homolog Hobit (homolog of Blimp1 in T cells) encoded by *znf683*, represent key transcriptional factors that control the development and differentiation of both B and T cells. Despite their essential role in the regulation of acquired immunity, this gene family has been largely unexplored in teleosts to date. Until now, one *prdm1* gene has been identified in most teleost species, whereas a *znf683* homolog has not yet been reported in any of these species. Focusing our analysis on rainbow trout (*Oncorhynchus mykiss*), an *in silico* identification and characterization of *prdm1*-like genes has been undertaken, confirming that *prdm1* and *znf683* evolved from a common ancestor gene, acquiring three gene copies after the teleost-specific whole genome duplication event (WGD) and six genes after the salmonid-specific WGD. Additional transcriptional studies to study how each of these genes are regulated in homeostasis, in response to a viral infection or in B cells in different differentiation stages, provide novel insights as to how this gene family evolved and how their encoded products might be implicated in the lymphocyte differentiation process in teleosts.

## Introduction

Transcription factors containing zinc finger binding domains represent a large family of regulators that control several important biological processes in mammals. Among them, Blimp1 (B lymphocyte-induced maturation protein 1) and Hobit (homolog of Blimp1 in T cells), homologous to each other, play a critical role in the regulation of B and T cell functionality.

Blimp1, also known as PR domain zinc finger protein 1, encoded by the *prdm1* gene, was initially described as a repressor of the human interferon β gene (*ifnβ*), specifically binding the PRDI element of the *ifnβ* promoter (1). In a later study, Turner et al. evidenced that Blimp1 is a key transcriptional regulator of B cells, driving the maturation of B lymphocytes into immunoglobulin (Ig) secreting cells (2). Thus, the expression of Blimp1 in B cells increases as they differentiate to plasmablasts/plasma cells, reaching highest transcript levels in quiescent long-lived plasma cells (3, 4). In fact, the capacity to generate plasmablasts/plasma cells is lost in organisms in which *prdm1* has been deleted (5, 6). At a molecular level, Blimp1 acts as a transcriptional repressor of other key genes involved in functions of undifferentiated B cells, along with other molecules such as X-box binding protein 1 (Xbp1) and interferon regulatory factor 4 (IRF4), also known to be critical to acquire a plasma cell phenotype (7). For example, Blimp1 was shown to be involved in the repression of transcription factor *c-myc*, thus provoking the exit from the cell cycle programme, a characteristic of terminally differentiated plasma cells (8, 9). Blimp1 also represses the promoters of genes involved in functions of mature naïve B cells such as Pax5 (10), Bach2 (11), or Bcl6 (12). In return, some of these factors, such as Bach2 or Bcl6, inhibit plasma cell differentiation by repressing the *prdm1* promoter (11, 13). Therefore, the differentiation of B cells to plasma cells is controlled by a complex regulatory network in which Blimp1 acts as a key element.

However, Blimp1 functions are not exclusively restricted to B cells, as it also regulates the fate and functionality of a wide range of tissues through its repressor activities (14, 15), as well as that of other immune cells including T cells (16, 17) and dendritic cells (18, 19). Specifically, a similar regulatory axis to that described in B cells involving Blimp1 and Bcl6 is triggered throughout the differentiation of CD4^+^ and CD8^+^ T cells (20). Thus, CD4^+^ T cells require Bcl6 to differentiate to T follicular helper cells (20, 21), whereas Blimp1 is involved in the later stages of differentiation of effector CD4^+^ T helper cells, when cells are characterized by a high cytokine secretion capacity and limited or no proliferative activity (20). Similarly, Blimp1 has been shown to be a key factor regulating effector CD8^+^ T cell functions, proliferation, and conversion into memory cells (20, 22, 23). Again, the lack of Blimp1 has been associated with a reduced capacity to secrete effector molecules such as granzyme (22, 23). Altogether, these results have unexpectedly revealed a common regulatory system used by CD4^+^ T cells, CD8^+^ T cells, and B cells in which Blimp1 is always associated with a reduced proliferative potential and a high effector molecule production (20).

The discovery of Hobit, also known as ZNF683, is much more recent in comparison to Blimp1, but has rapidly acquired relevance after establishing its influence on important processes in a range of immune cells. In mammals, it is believed that *znf683*, the gene coding for Hobit, and its adjacent gene *aim1l* arose by duplication of the region covering *prdm1* and *aim1* (24). Thus, Hobit shares a very high homology with Blimp1 within the zinc finger region, but lacks the SET domain that characterizes Blimp1 and other members of the PRDM family (24). Hobit was initially described in mouse, where it was shown to be preferentially expressed by natural killer T cells (NKT cells), while its expression levels in T and B cells were much lower (25). Thus, Hobit controls the differentiation of thymic mature NKT cells in this species, as well as their capacity to secrete granzyme and IFNγ (25). Years later, Hobit was identified in humans, where its expression was shown to be confined to NK cells and effector-type CD8^+^ T cells, being absent in naive or memory CD8^+^ T cells (24). Interestingly, recent evidence has associated Hobit with the differentiation and localization of tissue-resident memory CD8^+^ T cells (T_RM_ cells) [reviewed in (26)]. These memory cells are specifically retained at the sites where the primary infection has occurred, ready to provide a faster and increased secondary immune response to reinfection within peripheral tissues. However, the regulation of T_RM_ cells is not exclusively carried out by Hobit, but also requires the cooperation of Blimp1 (27). These two factors recognize similar targets in the regulatory sequences of their target genes, suggesting overlapping roles in the transcriptional repression of genes associated with lymphocyte migration (27).

Different studies have identified *prdm*1 homolog genes in several teleost fish species such as zebrafish *Danio rerio* (28, 29), fugu *Takifugu rubripes* (30), medaka *Oryzias latipes* (31), Japanese flounder *Paralichthys olivaceus* (32), or Nile tilapia *Oreachromis niloticus* (33). In medaka, two different genes have been described, *prdm1a* and *prdm1b*, both of which show detectable expression during embryo development and in all the tissues including immune organs (31). Furthermore, a significant induction of *prdm1a* in zebrafish and medaka was observed in the liver in response to different immune stimuli (31). Similarly, LPS stimulation increased the levels of transcription of Blimp1 in different Nile tilapia tissues (33). In this species, Blimp1 transcript levels were also shown to be higher in IgM^+^ B cells than in the negative fraction (33). In rainbow trout, *Oncorhynchus mykiss*, Diaz-Rosales et al. designed primers to study Blimp1 transcription after the identification of a *prdm1* homolog (34). These sequence and primers have been used in diverse studies to certify the up-regulation of Blimp1 transcription in trout IgM^+^ B cells in response to interleukin 6 (IL6) (35) or BAFF (36) activation. Interestingly, a population of IgD^-^IgM^lo^ B cells (cells with no IgD and low levels of IgM on the cell membrane) was identified in the rainbow trout peritoneal cavity, that seems to resemble antibody-secreting cells, in contrast to IgD^+^IgM^hi^ B cells (cells with IgD and high levels of IgM on the cell membrane) that correspond to naïve B cells (37). This antibody-secreting cell population also showed higher levels of transcription of this *prdm1* gene. Conversely, a Hobit*/znf683* gene has not yet been identified to date in teleost fish.

In the current study, a global analysis of *prdm1* homolog genes has been undertaken to investigate how this gene family has evolved. Our results demonstrate that the expansive evolution of these genes in teleost fish has resulted in a repertoire of six *prdm1*-like genes, four of them being likely homologs of mammalian *prdm1* and two of them homologs of *znf683*. All six genes were constitutively expressed in a wide range of tissues, and were up-regulated in response to a viral infection, whereas only three of them showed higher mRNA levels in peritoneal B cells with an Ig-secreting profile. These results point to an increased complexity of the molecular regulation of lymphocyte differentiation and fate in comparison to mammals and represent an important advance towards a better understanding of the mechanisms that drive lymphocyte differentiation in teleost fish.

## Material and Methods

### *In Silico* Identification and Analysis of *O. mykiss* blimp1-Like Genes

Using human the Blimp1 (Prdm1) and Hobit (Znf683) proteins as query and the tBLASTn software all potential *prdm1*-like genes from *O. mykiss* were identified in the two genomes available for this species, RefSeq Omyk_1.0 (considered reference genome) and Genoscope AUL_PRJEB4421_v1. Both transcripts and deduced amino acid sequences identified in each genome were compared to each other in order to filter the unique high confidence locus for the species. The same procedure was followed to identify all potential Prdm1-like proteins from *O. mykiss* present in UniProtDB. The presence and distribution of conserved domains in human Prdm1 and Znf683 proteins and in all *O. mykiss* Prdm1-like proteins were analyzed using InterProDB. Conserved regulatory elements were identified along the promoter regions from each gene. For this purpose, the first 1,000 bp upstream of start codon for each gene were extracted from human or rainbow trout RefSeq genomes and analyzed using the match tool of Transfac (database release 2019.2). Parameters were adjusted to compare with matrices from vertebrates using cut-offs to minimize false positive matches.

### Evolution of *blimp1* Genes Inferred From Phylogenetic Tree and Synteny Analysis

In order to analyze the evolution of *prdm1* genes, several protein sequences homologous to human Prdm1 or Znf683 from several species, as well as their genome context for synteny analysis, were retrieved from genomes present in the RefSeq database from NCBI. For this purpose, human Prdm1 or Znf683 proteins were used as queries against proteins included in different genomes from RefSeq database. Proteins from several species covering different classes were retrieved and included in a multiple protein alignment using ClustalW software. The alignment was used for the construction of a phylogenetic tree using maximum likelihood, testing the tree with a bootstrap method using 1,000 replications, all steps implemented in MEGA X software (38). Prdm1 proteins identified in *Branchiostoma belcherii* and *B. floridae* were included as outgroup and used for rooting purposes.

Synteny analysis was performed using the information related to *prdm1*-like genes and their gene neighboring genes available in RefSeq genomes from a set of key species. For this purpose, the most similar protein to each *prdm1* rainbow trout protein for a set of key species was identified in the phylogenetic tree previously constructed. The selection of species included several tetrapods (*Homo sapiens*, *Mus musculus*, *Gallus gallus, Nipponia nippon, Haliaeetus leucocephalus, Falco peregrinus, Podarcis muralis, Rhinatrema bivittatum, Chelonia mydas*, and *Xenopus tropicalis*), the chondrocyte elephant shark (*Callorhinchus milii*), different ancient fish considered in a key position during fish evolution (*Latimeria cholumnae*, *Erpetoichthys calabaricus*, and *Lepisosteus oculatus*) and several teleost fish (*Danio rerio, Esox lucius*, *Oreochromis niloticus*, *Oryzias latipes*, *Larimichthys crocea*, and *Takifugu rubripes*). Finally, a group representing salmonids (*Oncorhynchus mykiss*, *Salmo salar*, *Salmo trutta*, and *Salvelinus alpinus*) was also included in analysis. For all *prdm1* genes, the information related to the 5-6 coding neighboring genes at both sides to gene of interest were analyzed using the NCBI genome data viewer tool (https://www.ncbi.nlm.nih.gov/genome/gdv/), extracting the direction relative to *prdm1*, and the annotation assigned by RefSeq. The annotation was revised using BLASTp software comparing the coding protein sequence from neighboring genes as query against UniProtDB as reference database. To obtain the synteny images, the gene information was shown together with a phylogenetic tree based on the one previously constructed by Berthelot et al. (39).

### Experimental Fish

Healthy rainbow trout (*Oncorhynchus mykiss*) adults of approximately 100 g were obtained from *Piscifactoria Cifuentes* (Cifuentes, Guadalajara, Spain) and maintained at the animal facilities of the Animal Health Research Center (CISA-INIA, Spain) in an aerated recirculating water system at 16°C, with a 12:12 h light:dark photoperiod. Fish were fed twice a day with a commercial diet (Skretting) and were acclimatized to laboratory conditions for at least 2 weeks prior to any experimental procedure. During this period no clinical signs were ever observed.

### RNA Extraction From Tissues Obtained From Naïve Rainbow Trout

Fish were anaesthetized with benzocaine (Sigma). Blood was extracted with a heparinized needle from the caudal vein. Prior to sampling, a transcardial perfusion was conducted to remove all circulating blood from tissues. For this, the heart was cannulated through the ventricle into the bulbus arteriosus with approximately 30 ml of 0.9% NaCl, using a peristaltic pump (Selecta, Spain), while the atrium was cut to drain the blood out of the circulatory system. After perfusion, tissues (spleen, head kidney, posterior kidney, skin, gill, liver, thymus, and hindgut) were collected and placed in Trizol for subsequent RNA isolation.

Total RNA was extracted from tissue samples using a combination of Trizol (Invitrogen) and RNAeasy Mini kit (Qiagen) previously described (40). Briefly, samples were mechanically disrupted in 1 ml of Trizol using a disruption pestle. Then, 200 µl of chloroform were added and the suspension was centrifuged at 12,000 x *g* for 15 min. The clear upper phase was recovered, mixed with an equal volume of 100% ethanol and immediately transferred to RNAeasy Mini kit columns. The procedure was then continued following manufacturer’s instructions, performing on-column DNase treatment. Finally, RNA was eluted from the columns in RNase-free water, quantified in a Nanodrop 1000 spectrophotometer (Thermo Scientific) and stored at -80 °C until use. One µg of total RNA for each tissue was used to synthesize cDNA using the RevertAid Reverse Transcriptase (Thermo Scientific), primed with oligo(dT)_23_VN (1.6 µM), following the manufacturer’s instructions. The resulting cDNA was diluted in a 1:5 proportion with water and stored at -20 °C.

### Evaluation of *prdm1* Gene Transcription by Real Time PCR

To evaluate the levels of transcription of different *prdm1* genes, real-time PCR was performed in a LightCycler 96 System instrument (Roche) using FastStart Essential DNA Green Master reagents (Roche) and specific primers designed using the mRNA sequence from each *prdm1* gene (**Table 1**) and the Primer3 software (41). Each sample was subjected, in duplicate, to an initial cycle of denaturation (95°C for 10 min), followed by 40 amplification cycles (95°C for 10 s, 60°C for 10 s, and 72°C for 10 s). A dissociation curve was obtained by reading fluorescence every degree between 60 and 95°C to ensure only a single product was amplified. Negative controls with no template and *minus* reverse transcription controls (-RT) were included in all experiments. Gene expression was normalized to the relative expression of the rainbow trout elongation factor (EF-1α) amplified using primers previously used (42, 43). Expression levels were calculated using the 2^-ΔCt^ method, where ΔCt is determined by subtracting the reference gene value from the target Ct as described previously (42, 43).

**Table 1:** Primers used in real time analysis.

Gene ID	Gene name	Forward (5′–3′)	Reverse (5′–3′)
LOC110514303	*prdm1a-1*	CAGCGCCCCAGTCAAGATA	GGGGGTAGAGGGCACAGC
LOC110486999	*prdm1a-2*	CATTCGGCCCTATGTGTGG	CCCCTCGGTAGTCAACATGG
GSONMG00047229001	*prdm1b-1*	ACGACGTCATCGCACACTTC	CCCTCCCCAAACGGGTAA
LOC110496128	*prdm1b-2*	GGCTAGCGTGGTACGCTTCT	ACAAGGGTCCGTCCACTTTG
LOC110529181	*prdm1c-1*	TCACTGCATCAACACCGAGA	CCGGTCTCCATCACCATCTT
LOC110522002	*prdm1c-2*	CGCCAATGGGAATATGTCACT	GACATAGCCAGGATGCAGAGG

### Analysis of VHSV *In Vivo* Infection

Rainbow trout were infected with viral hemorrhagic septicemia virus (VHSV) using the 0771 strain which was propagated in the RTG-2 rainbow trout cell line as previously described (44). For this, rainbow trout were divided in two groups of 20 trout each. Groups were injected intraperitoneally with either 100 µl of culture medium (mock-infected control) or 100 µl of a viral solution containing 1 x 10^6^ TCID_50_ (tissue culture infective dose per ml). At days 1, 3, and 7 post-injection, six trout from each group were sacrificed by overexposure to benzocaine, and head kidney and spleen sampled and placed in Trizol. RNA was isolated and cDNA was obtained as described above for tissues obtained from naïve fish. The levels of transcription of the different *prdm1* genes were evaluated through real time PCR as described above.

### Isolation of Leukocyte Populations

The levels of transcription of the different *prdm1* genes were also evaluated in PBLs. For this, blood extracted from the caudal vein of anaesthetized rainbow trout was diluted 10 times with using Leibovitz’s medium (L-15, Gibco) containing 100 I.U./ml penicillin, 100 µg/ml streptomycin (P/S, Life Technologies), 10 U/ml heparin (Sigma- Aldrich), and 5% fetal calf serum (FCS). PBLs were then isolated placing blood samples onto 51% Percoll (GE Healthcare) density gradients. After centrifugation at 500 x *g* for 30 min at 4 °C, the interface cells were collected and washed with L-15 supplemented antibiotics and 5% FCS. Trizol was then used to obtain RNA following the manufacturer´s instructions and the levels of transcription of the different *prdm1* genes were evaluated through real time PCR as described above.

Leukocyte suspensions were obtained were obtained from the peritoneal cavity by perfusion with 2 ml of L-15 media supplemented with antibiotics, heparin and 5%FCS as described before (45). The cell suspension was then passed through a 100 µm nylon mesh (BD Biosciences) using L-15 medium supplemented with antibiotics, 10 U/ml heparin and 5% FCS and placed onto 30/51% Percoll discontinuous density gradients. After centrifugation of the gradients at 500 x *g* for 30 min at 4 °C, the interface cells were collected and washed with L-15 supplemented antibiotics and 5% FCS. The viable cell concentration was determined by Trypan blue (Sigma-Aldrich) exclusion and cells resuspended in L-15 with 5% FCS at a concentration of 1x10^6^ cells/ml. Cells with a plasmablast/plasma cell profile (IgM^+^IgD^-^ B cells from the myeloid gate) and naïve B cells (IgM^+^IgD^+^ B cells from the lymphoid gate) were isolated from peritoneal leukocyte populations as previously described (37). For this, peritoneal leukocytes were incubated with anti-IgM and anti-IgD specific monoclonal antibodies in staining buffer (phenol red-free L-15 medium supplemented with 2% FCS) for 1 h at 4°C. The anti-trout IgM [1.14 mAb mouse IgG_1_ coupled to R-phycoerythrin (R-PE), 1 µg/ml] and the anti-trout IgD [mAb mouse IgG_1_ coupled to allophycocyanin (APC), 5 µg/ml] used in this study have been previously characterized (46, 47) and were fluorescently labeled using R-PE or APC Lightning-Link labeling kits (Innova Biosciences) following manufacturer’s instructions. After the staining, cells were washed twice with staining buffer and FACS sorted using a BD FACSAria III (BD Biosciences). Only those samples showing a purity level higher than 95% were used for RNA isolation.

Splenic IgM^+^ B cells and CD8^+^ cytotoxic T cells were isolated from spleen as previously described (48). For this, single cell suspensions from spleen were prepared using 100 μm nylon cell strainers (BD Biosciences) and L-15 medium supplemented with antibiotics and 5% FCS. Cell suspensions were then placed onto 30/51% Percoll discontinuous density gradients and interface cells were collected and prepared as described above for peritoneal leukocytes. Spleen leukocytes were then labeled with anti-IgM (46) and anti-CD8 (49) specific antibodies as described above and IgM^+^ B cells and CD8^+^ T cells FACS sorted using a BD FACSAria III.

### Evaluation of *prdm1* Gene Transcription in Sorted Cell Populations

Total RNA was isolated from sorted cell populations using the Cells-to-Ct™ 1-Step Power SYBR™ Green Kit (Invitrogen) following manufacturer’s instructions as previously described (48). RNA was treated with DNase during the process to remove genomic DNA that might interfere with the PCR reactions. Reverse transcription was also performed using the Cells-to-Ct™ 1-Step Power SYBR™ Green Kit following manufacturer’s instructions. To evaluate the levels of transcription of the different genes, real time PCR was performed with a LightCycler^®^ 96 System instrument using SYBR Green PCR core Reagents (Applied Biosystems) and specific primers. Each sample was measured in duplicate under the following conditions: 10 min at 95 °C, followed by 40 amplification cycles (15 s at 95 °C and 1 min at 60 °C). A melting curve for each primer set was obtained by reading fluorescence every degree between 60 and 95°C to ensure only a single product had been amplified. The expression of individual genes was normalized to the relative expression of trout EF-1α as described above.

### Statistics

Data obtained from flow cytometry was analyzed using Microsoft Office Excel 2010 and GraphPad Prism version 7.00 for Windows (GraphPad Software). Statistical analyses were performed using a paired two-tailed Student´s *t* test. Statistical significance was considered significant on different degrees, where * means *P* ≤0.05, ** means *P* ≤0.01 and *** means *P* ≤0.001.

## Results

### Identification and Analysis of *Oncorhynchus mykiss* *blimp1* Homolog Genes

Human Blimp1 and Hobit protein sequences were used as queries against gene models from the two available rainbow trout genomes (Genoscope AUL_PRJEB4421_v1 and RefSeq Omyk_1.0) using tBLASTn searches. Similar results were obtained for both proteins. A total of six high confidence genes, annotated as “PR domain zinc finger protein 1-like” (*prdm1*-like) in RefSeq genome, were inferred from the analysis. The genes were located in different locus along the *O. mykiss* genome. In RefSeq Omyk_1.0, five genes are assigned to different chromosomes, whereas a group of four scaffolds were identified as a sixth gene not located in the genome. Using the previous genome from Genoscope, the six equivalent genes were also identified, considering that the four scaffolds identified in RefSeq Omyk_1.0 are equivalent to the gene GSONMG00047229001 from Genoscope. Further BLAST analysis using this gene along with its flanking regions were used as query using the RefSeq genome as reference. The results obtained seem to indicate that this gene is located in chromosome 14. A total of six Prdm1-like proteins deduced from the Genoscope genome were also identified in UniprotDB. Equivalences between datasets are shown in **Table 2**.

**Table 2:** *Prdm1* genes and proteins from different *O. mykiss* datasets.

Group	Name	*O. mykiss* RefSeq Genome V1.0											*O. mykiss* Genoscope Genome	
		Description	GeneID	Chr.	Chr. accession	Start position	End position	Orient.	Exon count	Symbol RefSeq genome	ACC RefSeq transcript	ACC RefSeq protein	ACC Genoscope genome	ACC protein Uniprot
*prdm1a*	*prdm1a-1*	PR domain zinc finger protein 1-like	110514303	3	NC_035079.1	26086965	26104101	Minus	8	LOC110514303	XM_021593764.1	XP_021449439.1	GSONMG00011799001	A0A060Y431
	*prdm1a-2*	PR domain zinc finger protein 1-like	110486999	2	NC_035078.1	16473063	16490152	Minus	8	LOC110486999	XM_021558900.1	XP_021414575.1	GSONMG00041848001	A0A060XWE1
*prdm1b*	*prdm1b-1*	PR domain zinc finger protein 1-like	110510852	Un	NW_018526823.1	–	–	–	–	LOC110510852	XM_021592048.1	–	GSONMG00047229001	A0A060WUV8
		PR domain zinc finger protein 1-like	110512408	Un	NW_018539670.1	–	–	–	–	LOC110512408	XM_021592452.1	–		
		PR domain zinc finger protein 1-like	110516101	Un	NW_018574236.1	–	–	–	–	LOC110516101	XM_021594702.1	–		
		PR domain zinc finger protein 1-like	110511601	Un	NW_018533479.1	–	–	–	–	LOC110511601	XM_021592816.1	–		
	*prdm1b-2*	PR domain zinc finger protein 1-like	110496128	18	NC_035094.1	30234061	30247025	Minus	8	LOC110496128	XM_021571829.1	XP_021427504.1	GSONMG00072073001	A0A060WI22
*prdm1c*	*prdm1c-1*	PR domain zinc finger protein 1-like	110529181	8	NC_035084.1	8351212	8362688	Minus	6	LOC110529181	XM_021611315.1	XP_021466990.1	GSONMG00011524001	A0A060Y1H9
	*prdm1c-2*	PR domain zinc finger protein 1-like	110522002	4	NC_035080.1	49630029	49646489	Minus	7	LOC110522002	XM_021600044.1	XP_021455719.1	GSONMG00019730001	A0A060XAE9

**Figure 1:**
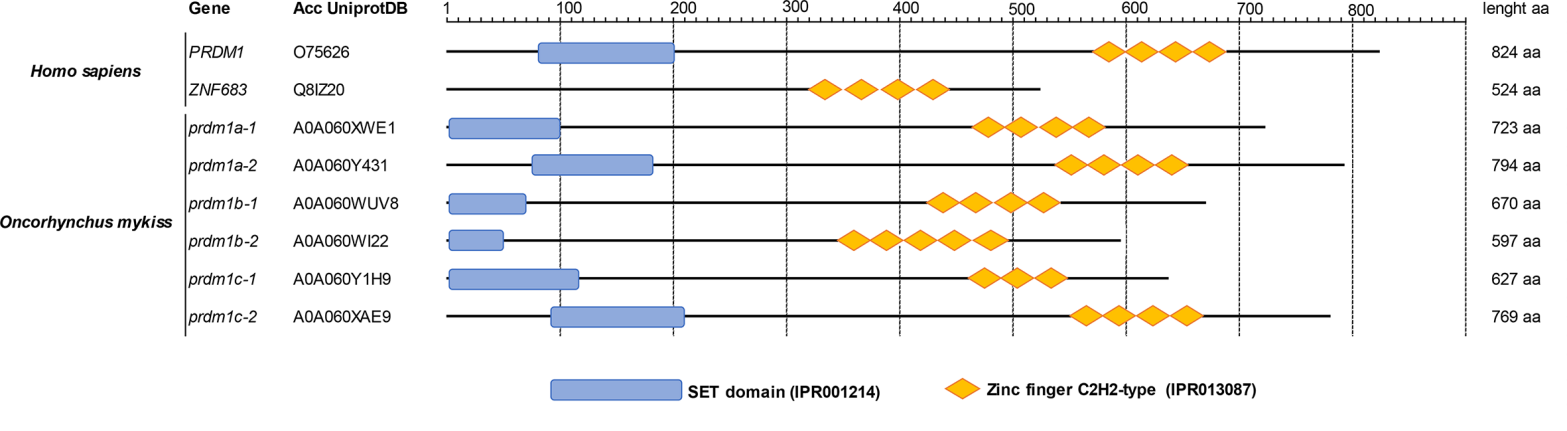
Analysis of conserved domains according InterPro database. The amino acid sequences of human PRDM1 and ZNF683 as well as those of Prdm1-like proteins from *O. mykiss* were compared with InterProDB to identify different conserved domains. All proteins showed homology with several tandem zinc finger C2H2-type domains at the C-terminus and a SET domain at the N-terminus with the exception of ZNF683, which does not present a SET domain.

An analysis of the conserved domains of all these *O. mykiss* Prdm1-like proteins was also carried out. In humans, Hobit is shorter than Blimp1 and does not contain a SET domain in the N-terminus region present in Blimp1 (**Figure 1**). In rainbow trout, all six Prdm1-like proteins contained a SET domain as well as the zinc finger C2H2-type domain characteristic of this gene family (**Figure 1**).

### Evolution of *prdm1* Genes Inferred From Phylogenetic Tree and Synteny Analysis

**Figure 2:**
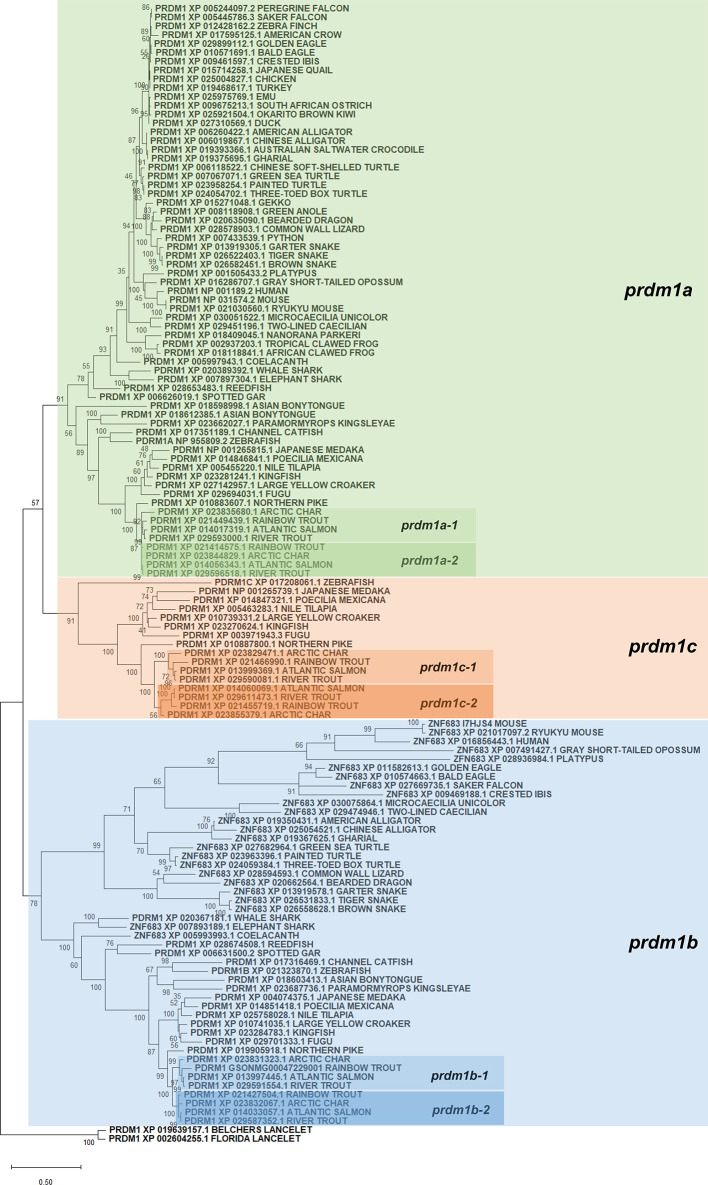
Phylogenetic tree using PRDM1 and ZNF683 proteins from the genomes of several species included in the RefSeq databases. Protein alignment was performed using the ClustalW software and a phylogenetic tree constructed using maximum likelihood analysis. Tree confidence was tested using bootstrapping analysis (1,000 replicates). Prdm1 proteins identified in *Branchiostoma belcherii* and *B. floridae* were included as outgroup and used for rooting purposes. The tree constructed highlights the three groups of *prdm1* genes identified in fish species (*prdm1a*, *prdm1b*, and *prdm1c*). Names assigned to different *prdm1* genes from salmonids are also included in the phylogenetic tree.

Protein sequences annotated as Prdm1 or Znf683 from several species (**Supplementary Table S1**) as well as their genome context were retrieved from genomes present in RefSeq databases from NCBI. Protein alignments were performed using ClustalW software and a phylogenetic tree constructed using maximum likelihood in order to analyze the evolution of *prdm1* genes (**Figure 2**). According to this analysis, all *prdm1*-like genes have evolved from an ancestral gene that suffered different duplication events which resulted in an increase of gene copies in different groups during evolution. In lancelets, represented by *Branchiostoma floridae* and *B. belcheri*, both used as outgroup in the phylogenetic analysis, only one gene homolog to *prdm1* was identified. This ancestral gene seems to have experienced a first duplication event before the emergence of chondrocytes which is reflected by the presence of two gene copies both in whale shark (*Rhincodon typus*) and elephant shark (*Callorhinchus milii*). This first duplication node resulted in two differentiated groups which include mammalian *prdm1* and *znf683*, respectively. In fish, these genes have been designated as *prdm1a* (closely related to mammalian *prdm1)* and *prdm1b* (closely related to mammalian *znf683)* following the zebrafish RefSeq annotation (**Figure 2**). In addition to chondrocytes, human, and mouse, homologs of these two genes are also present in coelacanth (*Latimeria chalumnae*) and several snakes, reptiles, and birds. The analysis performed further shows that after the emergence of tetrapods, the gene corresponding to *prdm1a* was yet again duplicated in teleost fish. Thus, several teleost fish from different classes show an additional *prdm1*-like homolog designated as *prdm1c* that seems to have appeared as a consequence of *prdm1a* duplication (**Figure 2**). This additional gene is not present in ancient fish such as reedfish (*Erpetoichthys calabaricus*) or spotted gar (*Lepisosteus oculatus*). Finally, the phylogenetic analysis also shows that these three genes present in teleost were further duplicated in salmonids, resulting in six *prdm1*-like genes. This duplication was evident in rainbow trout and other salmonids included in analysis such as Atlantic salmon (*Salmo salar*), river trout (*Salmo trutta*), and arctic char (*Salvelinus alpinus*). In salmonids, the genes resulting after the gene duplication of *prdm1a*, *prdm1b*, and *prdm1c* were identified as -1 or -2 (**Figure 2**, **Table 2**).

**Figure 3:**
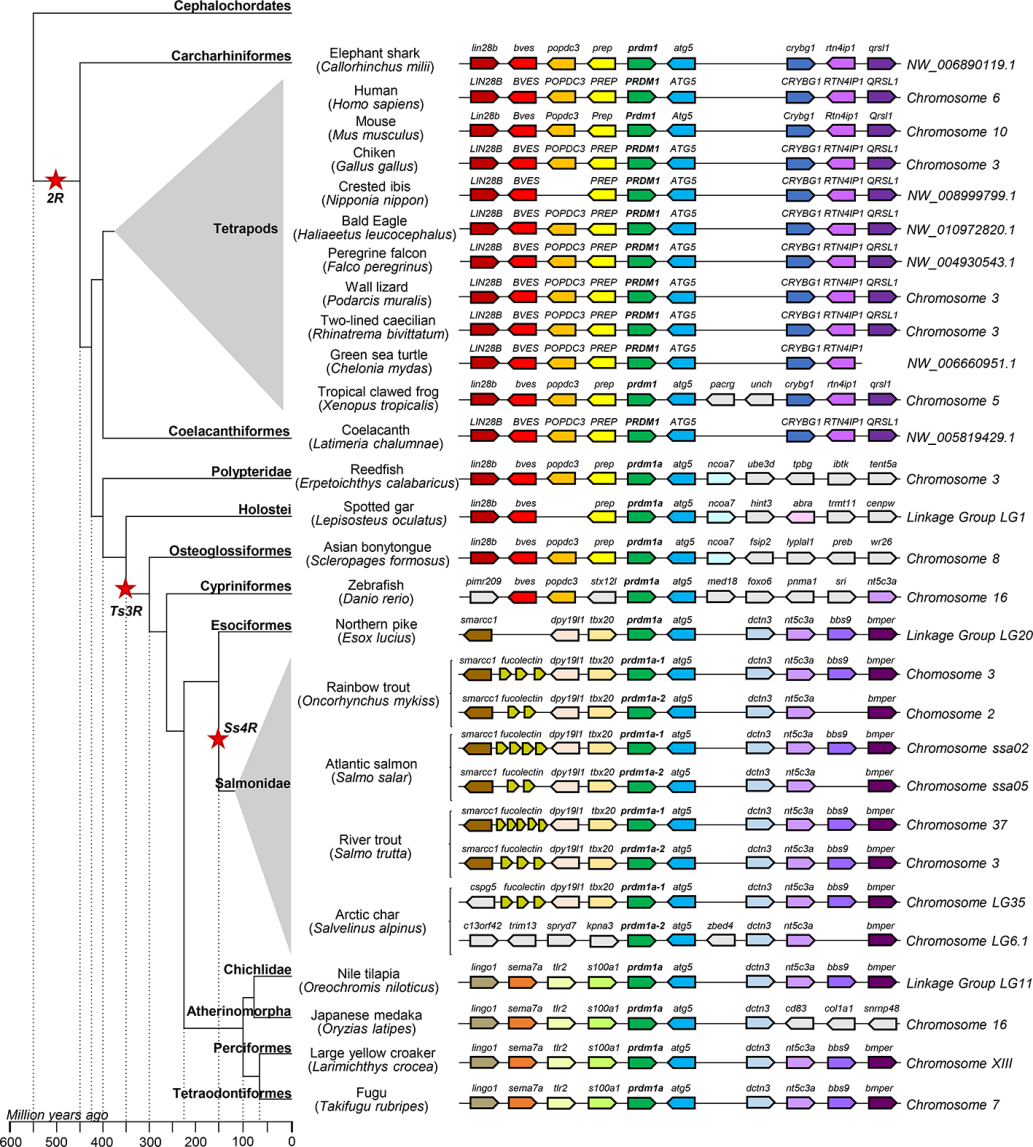
Synteny analysis of the *prdm1a* group from *O. mykiss* associated to phylogenetic reconstruction. Different genes are represented by different colored arrows. The *prdm1a* neighboring genes were identified along different genomes available in the RefSeq database. Nonconserved genes along the figure are shown in gray. Arrows indicate the gene direction relative to *prdm1a*.

In order to verify the evolution inferred from the phylogenetic analysis performed, the genome context of the different homolog genes was analyzed. *Prdm1a* showed remarkable synteny conservation among tetrapods, also conserved in elephant shark. The synteny is also totally preserved in coelacanths and partially conserved in the ancient fish *E. calabaricus* and *L. oculatus*. After the teleost-specific duplication event, the synteny is only slightly preserved in zebrafish and practically reduced to the conservation of the nearest gene, *atg5*, in *Esox lucius* and all salmonids. Remarkably, the synteny conservation between esociformes and salmonids is really high. Additionally, high synteny conservation was observed between the two *prdm1a* genes in each salmonid species included in the analysis with the exception of *S. alpinus* that showed a lower degree of synteny conservation. On the contrary, *O. mykiss* and *S. salar* only presented slight differences between the synteny of the two *prdm1a* genes in a region where a variable number of *fucolectin* genes were identified, probably as result of recent tandem duplications (**Figure 3**).

**Figure 4:**
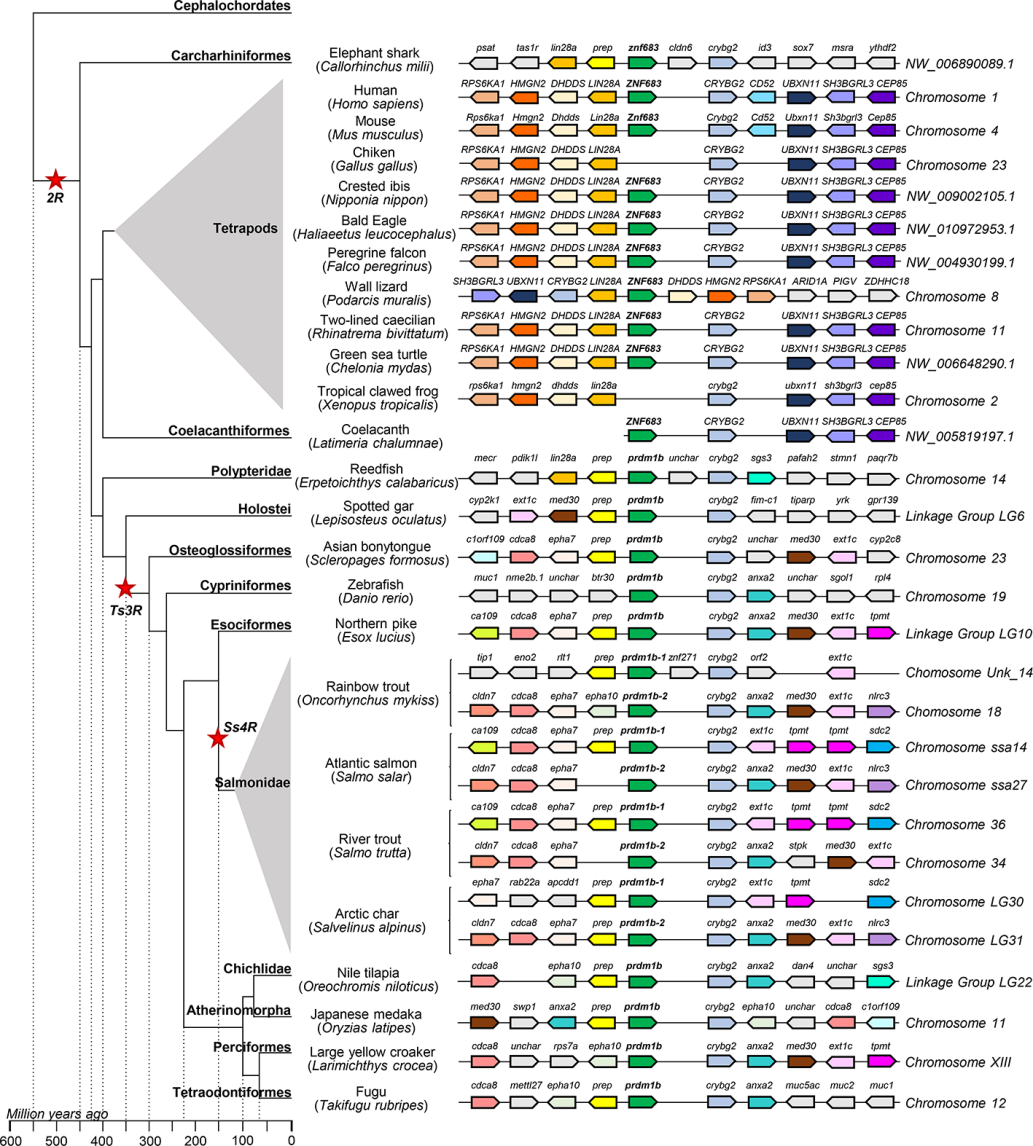
Synteny analysis of the *prdm1b* group from *O. mykiss* associated to phylogenetic reconstruction. Different genes are represented by different colored arrows. The *prdm1b* neighboring genes were identified along different genomes available in the RefSeq database. Nonconserved genes along the figure are shown in gray. Arrows indicate the gene direction relative to *prdm1b*.

*Prdm1b* is present in the genome of elephant shark and coelacanth according to the phylogenetic analysis previously performed. The search for these genes in the RefSeq database showed that both genes were annotated as *znf683*. Interestingly, synteny analysis highlighted a total conservation between this gene region in coelacanth and the *znf683* gene from human pointing to this gene as an ancestor of mammalian *znf683* (**Figure 4**). Similarly, total synteny conservation was observed in mouse (**Figure 4**). Although in mouse, *znf683* appears as a pseudogene in the genome, based on the information contained within the databases, the expression of this gene has been previously verified (25). This gene seems to be absent in the genome of *G. gallus* or *X. tropicalis* despite the fact that other genes within this region are well conserved in these species. In the case of elephant shark, partial synteny conservation was observed between the *blimp1b* region and the human and coelacanth genomes, as it retained the *crybg2* and *lin28a* genes (**Figure 4**). Furthermore, a set of genes that appears downstream *znf683* in human and mouse (*ubxn11*, *sh3bgrl3*, and *cep85*) translocated upstream *znf683* in the elephant shark genome (data not shown). Despite this, in the elephant shark genome, the *prep* and the *crybg2* gene are located relative to *znf683* in a similar position to that observed in reedfish and most teleosts (**Figure 4**). The zebrafish *rpdm1b* region maintains *crybg2* but has lost the *prep* gene (**Figure 4**). In the case of salmonids, the synteny conservation between the two *rpdm1b* genes is low as one of these genes usually retains the *prep* gene upstream *rpdm1b* along with the *crybg2* gene, whereas the other copy contains the *anxa2*, the *med30* and the *ext1c* genes downstream the *rpdm1b* along with the *crybg2* gene (**Figure 4**).

**Figure 5:**
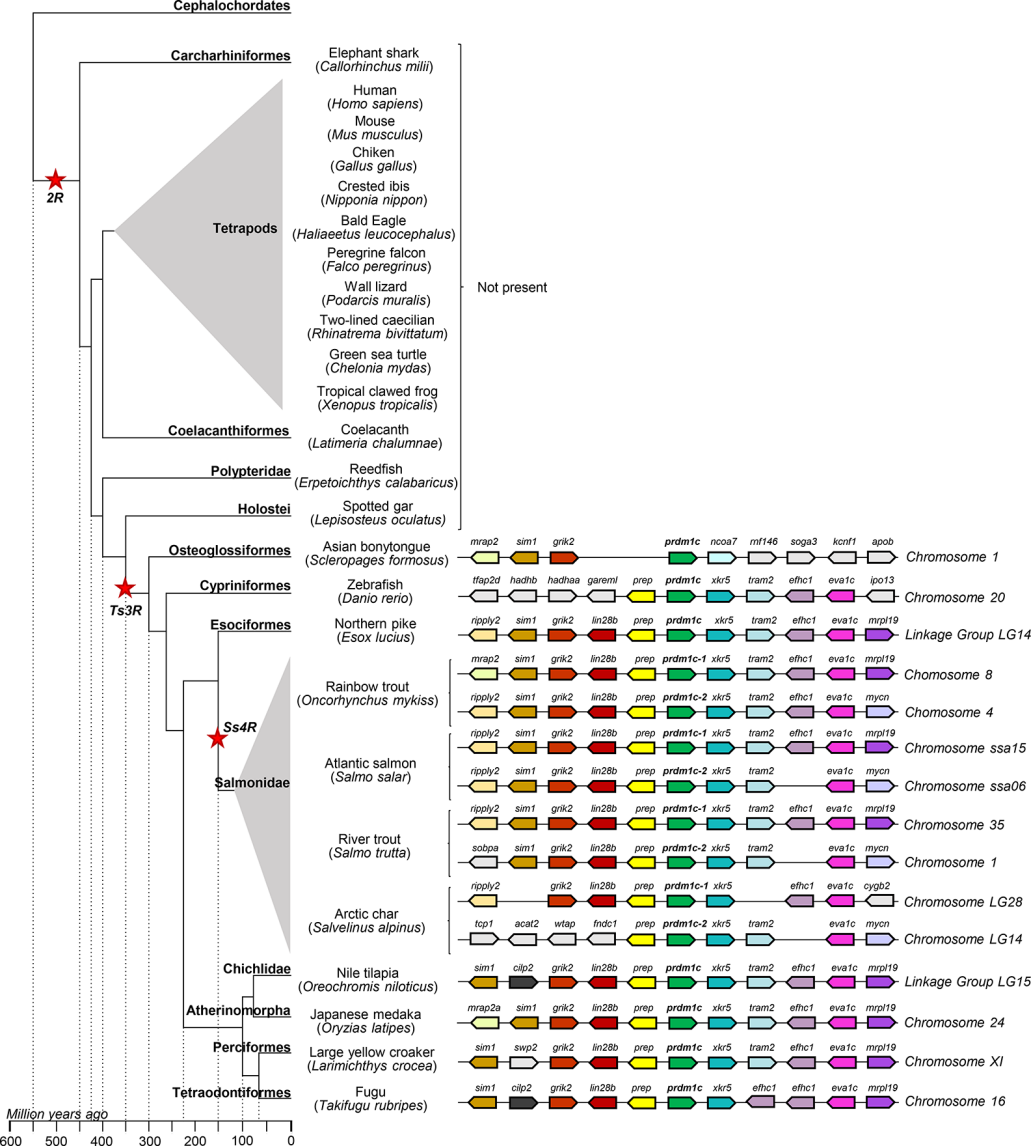
Synteny analysis of the *prdm1c* group from *O. mykiss* associated to phylogenetic reconstruction. Different genes are represented by different colored arrows. The *prdm1c* neighboring genes were identified along different genomes available in the RefSeq database. Nonconserved genes along the figure are shown in gray. Arrows indicate the gene direction relative to *prdm1c*.

Finally, *prdm1c* is only present in teleost fish. The degree of synteny conservation in the *prdm1c* region is almost total between the *E. lucius* and salmonids, and is also highly conserved between both genes found in the salmonid genomes (**Figure 5**). The degree of synteny conservation regarding zebrafish gene is lower but also significant. Interestingly, the genome region of *prdm1c* contains the *prep* gene in the same position and direction than the observed in the *prdm1a* region in tetrapods and ancient fish and in the *prdm1b* region in elephant shark and ancient fish (**Figure 5**). The fact that the *prep* gene is found associated to all *prdm1a, b*, and *c* seems to further support a common origin for all *prdm1*-like genes.

Thus, the synteny analysis of these three genomic regions supports the phylogenetic analysis previously performed and point to *prdm1a* and *prdm1c* genes as ancient homologs of mammalian *prdm1* and *prdm1b* genes being in fact an ancient homolog of mammalian *znf683* genes (**Figures 3**–**5**).

### Analysis of Promoter Regions in *prdm1*-Like Genes

An analysis of regulatory elements in the promoter region was also undertaken for the six rainbow trout *prdm1*-like genes. In this case, a high variability between the different rainbow trout promoter sequences was observed (**Supplementary Figure S1**). A set of regulatory elements are highly represented along the promoters of all *prdm1*-like genes including binding domains for Znf333, Klf6, Sox, or NFAT related factors. Interestingly, the human *prdm1* gene contains a regulatory element for Bcl6 factors which has been experimentally validated (50). This element was identified in the promoter region of rainbow trout *prdm1a-1* and *prdm1c-1*, likely homologs of mammalian *prdm1*. Notably, both *blimp1a-2* and *blimp1b-1* genes contain in their promoter regions a binding domain for Blimp1 that point to a feedback regulatory mechanism or to a control of the expression of these genes by other Prdm1-like proteins. Finally, rainbow trout *prdm1b-1* also contains different regulatory elements for p53 related factors in the promoter region, as human *znf683* (**Supplementary Figure S1**).

### Constitutive Expression Analysis of *O. mykiss* *blimp1* Genes

**Figure 6:**
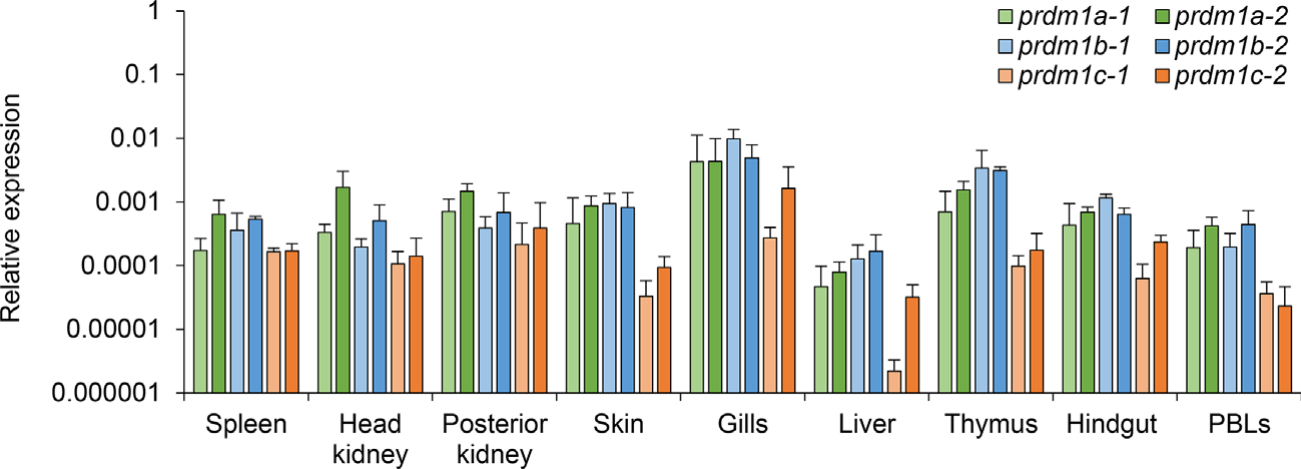
Constitutive expression analysis of *O. mykiss* *prdm1* genes. The levels of transcription of different *prdm1* genes were analyzed in several tissues/cells obtained from healthy fish including the spleen, head kidney, posterior kidney, skin, gill, liver, thymus, hindgut, and PBLs. Gene expression data were normalized against the endogenous control *ef-1α* and are shown as relative expression levels (means + SD; n = 3).

We next studied the levels of transcription of the six *prdm1* genes identified in the *O. mykiss* genome in nine different tissues/cells obtained from naive fish including spleen, head kidney, posterior kidney, skin, gill, liver, thymus, hindgut, and PBLs (**Figure 6**). Real time PCR analysis confirmed that all *O. mykiss* *prdm1* genes were constitutively transcribed in all rainbow trout tissues studied. In general, all genes showed similar transcription patterns along different organs showing the highest expression in gills and lowest expression in liver (**Figure 6**). Slight differences were observed between the transcription levels of the different *prdm1* subgroups. For example, *prdm1a-2* commonly showed higher transcript levels than other *prdm1*-like genes in B cell-rich immune organs, such as spleen, head and posterior kidney (**Figure 6**). In contrast, the *blimp1b* genes present higher transcription levels in homeostasis in mucosal tissues such as skin, gills, and hindgut as well as in liver and thymus (**Figure 6**), tissues known to be rich in T cells (51). The two genes *blimp1c* always showed the lowest levels of constitutive transcription in all organs studied (**Figure 6**). It has to be taken into account that higher transcription values in specific tissues could be indicating that these sites contain a limited number of cells with very high expression levels or many cells with intermediate values.

### Expression Analysis of *O. mykiss* *blimp1* Genes During Viral Infection

**Figure 7:**
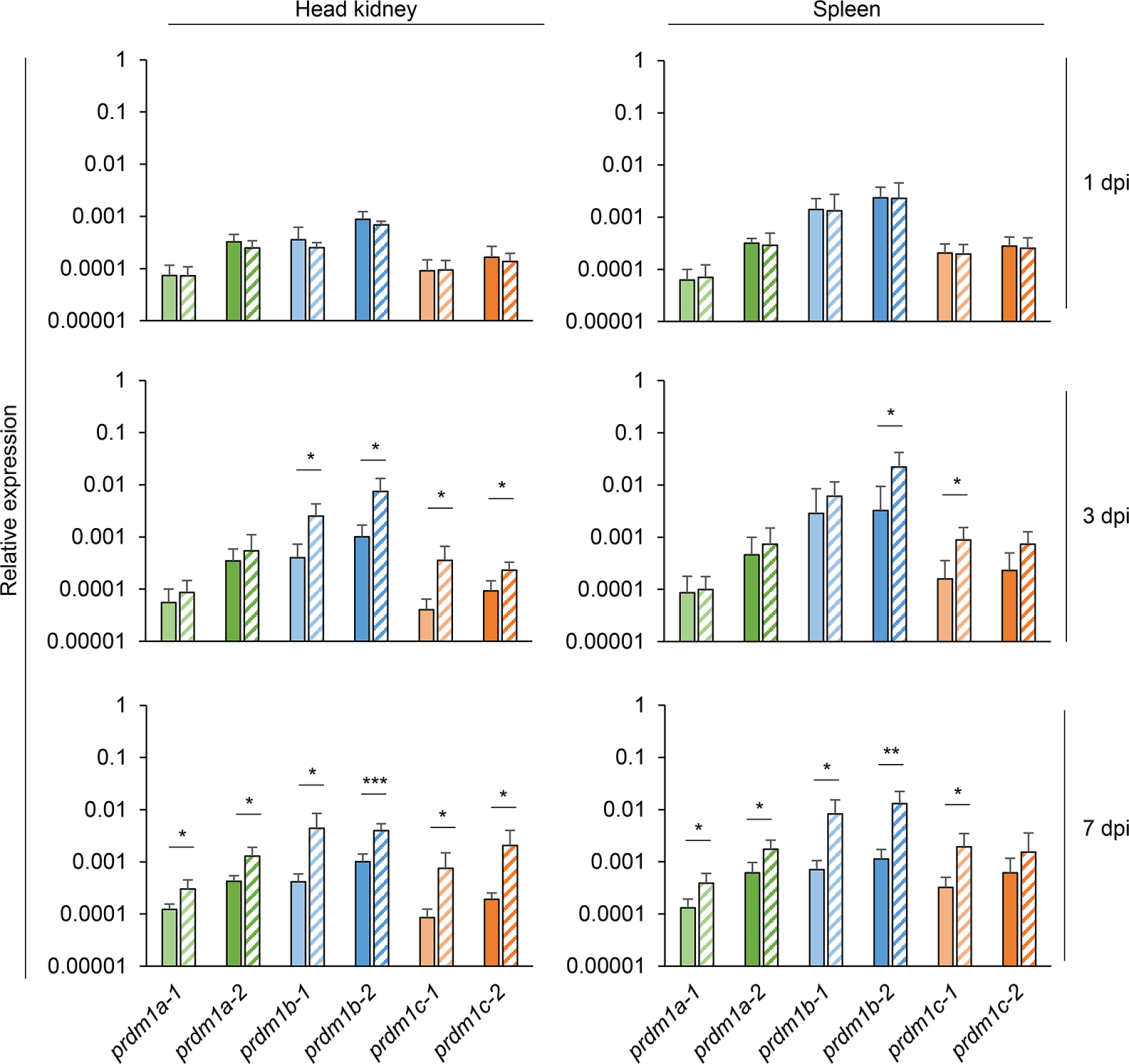
Transcriptional patterns of *O. mykiss* *prdm1* genes in head kidney and spleen during viral infection. The levels of transcription of the different *prdm1* genes were analyzed in the head kidney and spleen in fish infected with VHSV by intraperitoneal injection or mock-infected fish at days 1, 3, and 7 days post-infection. Gene expression data was normalized against the endogenous control *ef-1α* and is shown as relative expression levels (means + SD; n = 6). Asterisks indicate significant differences between transcription levels in VHSV-infected fish and control fish (*p < 0.05, **p < 0.01, and ***p < 0.005).

We next studied the transcriptional response of the six rainbow trout *prdm1* genes during a viral infection with a natural rainbow trout pathogen. For this, we determined the levels of transcription of these genes in head kidney and spleen of trout infected with VSHV by intraperitoneal injection and mock-infected controls at days 1, 3, and 7 post-infection. No significant changes were observed at day 1 post-injection for any of the genes (**Figure 7**). After 3 days of infection, the transcription levels of *prdm1b1, prdm1b2, prdm1c1*, *and prdm1c2* significantly increased in response to the viral infection in the head kidney, while only *prdm1b2* and *prdm1c1* were significantly augmented at this point in spleen (**Figure 7**). At day 7 post-infection, the transcription levels of all six *prdm1* genes significantly increased in response to VHSV in the head kidney (**Figure 7**). In the spleen, the transcription of all genes but *prdm1c2* were significantly upregulated in infected fish compared to controls (**Figure 7**). Again, in this case, it should be considered that up-regulation of *prdm1* transcription could be indicating an up-regulation of the gene in local cells or a recruitment of cells with higher transcription levels in response to the virus.

### Expression Analysis of *O. mykiss* *blimp1* Genes in Isolated B and T Cell Subsets

**Figure 8:**
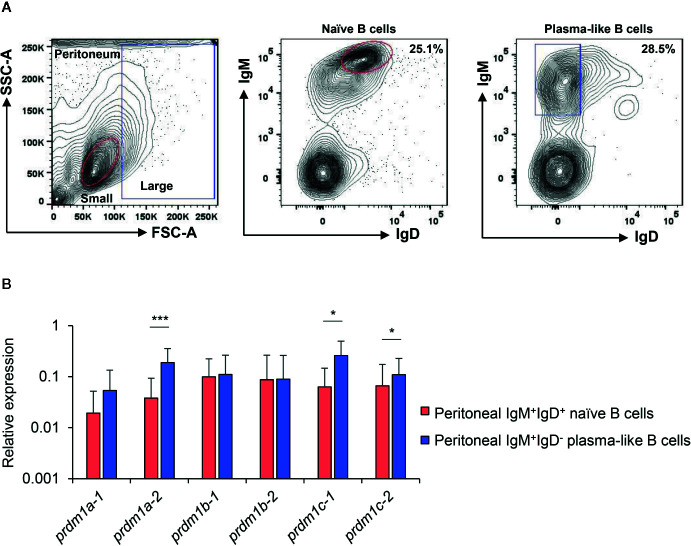
Expression analysis of *O. mykiss* *prdm1* genes in isolated B cell subsets from the peritoneum. Levels of transcription of all rainbow trout *prdm1* genes in different B-cell subsets from the peritoneum, including IgM^+^IgD^+^ naive B cells from lymphoid gate and IgM^+^IgD^−^ plasma-like cells from the myeloid gate **(A)** Representative plots showing sorted populations. Gene expression data were normalized against the endogenous control *ef-1α* and are shown as relative expression levels (means + SD; n = 10). **(B)** Asterisks indicate significant differences between transcription levels in the two B cell populations (*p < 0.05 and ***p < 0.005).

As mentioned above, in mammals, Blimp1, the product of *prdm1*, is a key transcriptional regulator of B cells that drives the maturation of B lymphocytes into Ig secreting cells (2), being an essential factor to generate long-lived plasma cells (3, 4). Previous studies from our group demonstrated that the peritoneal cavity of rainbow trout bears both IgM^+^IgD^+^ B cells within the lymphoid gate (small cells with low complexity) that correspond to naïve B cells and IgM^+^IgD^-^ B cells within the myeloid gate (large cells with higher complexity) that seem to resemble antibody-secreting cells (plasmablasts/plama cells) (37). These antibody-secreting cells were shown to have higher levels of a *prdm1* gene initially identified by Diaz-Rosales and collaborators (34). Based on the new nomenclature assigned now to the six *prdm1* genes identified in this work, this sequence corresponds to *prdm1c-2.* Thus, in the current work, we isolated again these two subpopulations and studied the levels of transcription of all six *prdm1* genes. All genes were transcribed by both subpopulations (**Figure 8**). As previously described (37), *prdm1c-2* mRNA levels were significantly higher in plasmablasts/plasma cells than in naïve cells. Similar results were also observed for *prdm1c-1*and specially for *prdm1a-2* genes (**Figure 8**), which also showed significantly higher transcription levels in plasmablasts/plasma cells than in IgM^+^IgD^+^ naïve B cells. Finally, we also determined whether these six *prdm1* genes were transcribed by splenic CD8^+^ T cells. In this case, we also isolated splenic naïve IgM^+^IgD^+^ B cells for comparative purposes. In contrast to what occurred in peritoneal naïve B cells, splenic naïve B cells only transcribed *prdm1a-2*, *prdm1b-1* and *prdm1c-1* constitutively (**Supplementary Figure S2**). However, splenic CD8^+^ T cells constitutively transcribed all six *prdm1* genes with higher transcription levels than those found in splenic naive B cells (**Supplementary Figure S2**).

## Discussion

Blimp1 (encoded by *prdm*1) and Hobit (encoded by *znf683*) are key regulators of several processes in mammals. In fish, different studies have identified homologs to mammalian *prdm1* genes (28–33), however to date, a full phylogenetic analysis of this gene family had not been undertaken in any fish species. In the current work, we have identified six different loci encoding functional genes belonging to this gene family in rainbow trout and other salmonid species. This discovery sheds light into how this gene family has changed throughout evolution as a result of genome duplications.

Previous analysis undertaken in human and mouse already suggested that *prdm1* and *znf683* genes evolved from a common ancestor gene, based on the similarities in their genomic context (24, 25). The phylogenetic analysis carried out in this work further supports this theory, by demonstrating the presence of two *prdm1* genes in the genome of the chondrocytes such as elephant or whale shark which indicates that the first gene duplication of *prdm1*-like genes occurred before the emergence of these species. Based on the phylogenetic analysis, these two resulting genes seem to be homologs of mammalian *prdm1* and *znf683*, respectively. Notably, while homologs of *prdm1* were identified in practically all species with genome in the RefSeq database, *znf683* homologs were only found in a reduced number of species. However, *prdm1a* genes that grouped together with mammalian *prdm1* genes and *prdm1b* genes that grouped with *znf683*, were found in all fish species analyzed. Remarkably, the branch lengths in the phylogenetic tree indicate a lower evolution rate in the *prdm1* group whereas a fast evolution is suggested for the *znf683* gene. This could be the reason why, in a set of species such as human and mouse, *znf683* lost its SET domain, possibly resulting in the neofunctionalization of this gene in these species. In other species, the fast evolution of this region might have resulted in the loss of the gene and its product. The synteny analysis performed with a group of representative salmonid species and a group of key species along evolution clearly corroborated these results. Thus, the *atg5* gene is neighboring *prdm1a* as it is for mammalian *prdm1*, conserving orientation in all species studied from elephant shark to salmonids. Similarly the *crybg2* gene (also named *aim1l*) is neighboring *prdm1b* in all species studied, as it is the case for human and mouse *znf683*.

Both the phylogenetic and the synteny analysis seem to indicate that *prdm1c* arose from a duplication of *prdm1a* specifically in teleosts, most probably during the teleost-specific whole genome duplication (WGD) (52). This hypothesis is supported by the absence of this third *prdm1* gene in coelacanths or ancient fish species like spotted gar. Finally, as a result of the salmonid-specific WGD, *prdm1* genes from salmonids represented by rainbow trout, Atlantic salmon, river trout and arctic char, suffered a further duplication of the three *prdm1* genes (*prdm1a*, *prdm1b*, and *prdm1c*) resulting in six different loci along the salmonid genomes. Interestingly, the synteny conservation between each pair of duplicated genes in salmonids is remarkable and reinforces the fact that the second copy for each *prdm1* gene appeared during this salmonid-specific WGD. Interestingly, the lower degree of synteny conservation between *prdm1b-1* and *prdm1b-2* again reflects that this genomic region is probably suffering higher evolutionary pressure than the other *prdm1* genes, in agreement with a fast evolution of *znf683* from an ancestral *prdm1b*.

After conducting the *in silico* analysis of this gene family, we performed several transcriptional analysis to provide insights on what the role of these six Prdm1-like proteins would be in salmonids using the rainbow trout as a model. A transcriptional analysis in tissues/cells obtained from naive fish, allowed us to confirm that all six *prdm1* genes are constitutively transcribed throughout a range of immune and mucosal tissues. Next, we determined whether the levels of transcription of these six genes were regulated in response to a viral infection in immune tissues. We performed this study in spleen as the main secondary organ in fish and in head kidney given that many studies have established in rainbow trout that this is a site where plasmablasts/plasma cells migrate to and are maintained for long time periods after stimulation (53–55). Our results demonstrated that all six genes were up-regulated in response to VHSV in the head kidney, whereas all of them but *prdm1c-2* did so in the spleen. These results strongly suggest that all six proteins have an important role in the rainbow trout immune response, possibly through the regulation of B and T cell functionality.

To study which of these genes are regulated in rainbow trout throughout the differentiation of B cells to plasmablasts/plasma cells, we examined the levels of transcription of these genes in peritoneal B cells, among which a population differentiated to plasmablasts/plasma cells had been previously characterized in rainbow trout (37). As established before (37), the transcription levels of *prdm1c-2* which corresponds to the *prdm1* gene previously identified by Diaz-Rosales and collaborators (34), are significantly higher in cells with a plasmablast/plasma cell profile (IgM^+^IgD^-^ B cells) than those observed in naïve B cells (IgM^+^IgD^+^ B cells). Interestingly, in our work, we found that *prdm1c-1*and especially *prdm1a-2* genes were also significantly higher in plasmablasts/plasma cells when compared to naïve cells. *prdm1a-1* mRNA levels were also higher in these cells, but in this case the differences with the levels observed in naïve cells were not significant. It is interesting that *prdm1b-1* and *prdm1b-2*, homologs of *znf683*, although expressed in both B cell populations did not seemed affected by the B cell differentiation process. However, our results demonstrate that in salmonids, unlike the situation in mammals, several Blimp1-like factors are induced during the B cell differentiation process. Thus, these factors could be interacting with one another through unknown mechanisms to regulate lymphocyte differentiation or instead it might be possible that each of these factors is involved in different stages of the differentiation process or are specific to some cell types. These possibilities should be further investigated in future studies. Furthermore, the fact that *prdm1c* genes, which arose as result of *prdm1a* duplication in teleost, also showed higher mRNA levels in plasmablasts/plasma cells from the peritoneum than naïve B cells supports that *prdm1c* genes have retained the functionality of mammalian *prdm1* genes implicated in lymphocyte differentiation.

In mammals, Znf683 is mainly implicated in the differentiation of T cells. To date, in rainbow trout, the tools to isolate differentiated T cell populations are not yet available, thus as an initial step we have examined the levels of transcription of the six *prdm1* genes in isolated splenic CD8^+^ T cells. Our data show that these T cells constitutively transcribe all six *prdm1* genes, however, whether all or some of these genes are up-regulated upon activation throughout the differentiation process is still unknown. In any case, it could be plausible that, as recently elucidated in mammals, Blimp1 and Hobit homologs cooperate in the regulation of the differentiation of both B and T cells, as transcripts for all six genes were detected in most B and T cell populations tested, probably with Blimp1 playing a preferential role in B cells and Hobit in T cells. Along with this hypothesis, it is worth noting that the levels of transcription of *prdm1b* genes was higher in tissues know to be rich in T cells such as gills, liver, thymus, and hindgut (51).

In conclusion, our results reveal a stepwise expansion of the *prdm1* family from a common ancestor to a repertoire of six functional genes present in salmonids. Among them, *prdm1a* and *prdm1c* genes seem to be closely related to mammalian *prdm1* whereas teleost *prdm1b* genes seem to be the ancestor of mammalian *znf683* genes. In any case, the preservation of all of these genes throughout evolution highlights that their encoded proteins carry out important functions in teleost fish. Thus, all six genes were constitutively transcribed in a wide range of tissues and in all cases their levels of transcription were significantly upregulated in response to a viral infection, pointing to an immune role. Finally, although the fact that all six genes were constitutively transcribed by peritoneal B cells and splenic CD8^+^ T cells suggests the implication of all of their products in the differentiation/homeostasis of B and T cells, at still unknown levels, only genes closely related to mammalian *prdm1* (*prdm1a-2*, *prdm1c-1*, and *prdm1c-2*) were found significantly up-regulated in plasmablasts/plasma cells, suggesting a preferential role of these Blimp1-like encoded proteins in the differentiation of B cells. These results provide us with a complete portrait of how this gene family evolved, also constituting a first step towards a full comprehension of the lymphocyte differentiation process in salmonids.

## Data Availability Statement

The raw data supporting the conclusions of this article will be made available by the authors, without undue reservation.

## Ethics Statement

The animal study was reviewed and approved by Ethics Committee from INIA (Code PROEX 002/17).

## Author contributions

PP performed sequence similarity searches, designed and performed phylogenetic analyses, and drafted the manuscript and figures with collaboration from SB. MG-E performed all the transcriptional experiments in collaboration with DM, IS, and PD-R. EM provided support with all flow cytometry experiments and performed the cell sortings. CT conceived the work, designed the experiments, and wrote the final version of the manuscript with help from PP and PD-R. All authors contributed to the article and approved the submitted version.

## Funding

This work was supported by the European Research Council (ERC Consolidator Grant 2016 725061 TEMUBLYM), by the Spanish Ministry of Science, Innovation and Universities (project AGL2017-85494-C2-1-R) and by the *Comunidad de Madrid* (grant 2016-T1/BIO-1672).

## Conflict of Interest

The authors declare that the research was conducted in the absence of any commercial or financial relationships that could be construed as a potential conflict of interest.
