# Supplementary material for: Insights Into the Evolution of the prdm1/Blimp1 Gene Family in Teleost Fish
Source: Front Immunol. 2020 Oct 30;11:596975. doi: 10.3389/fimmu.2020.596975 (PMC7662092; doi:10.3389/fimmu.2020.596975)
Supplement: Supplementary file 1 [file Presentation_1.pptx]

## Slide 1
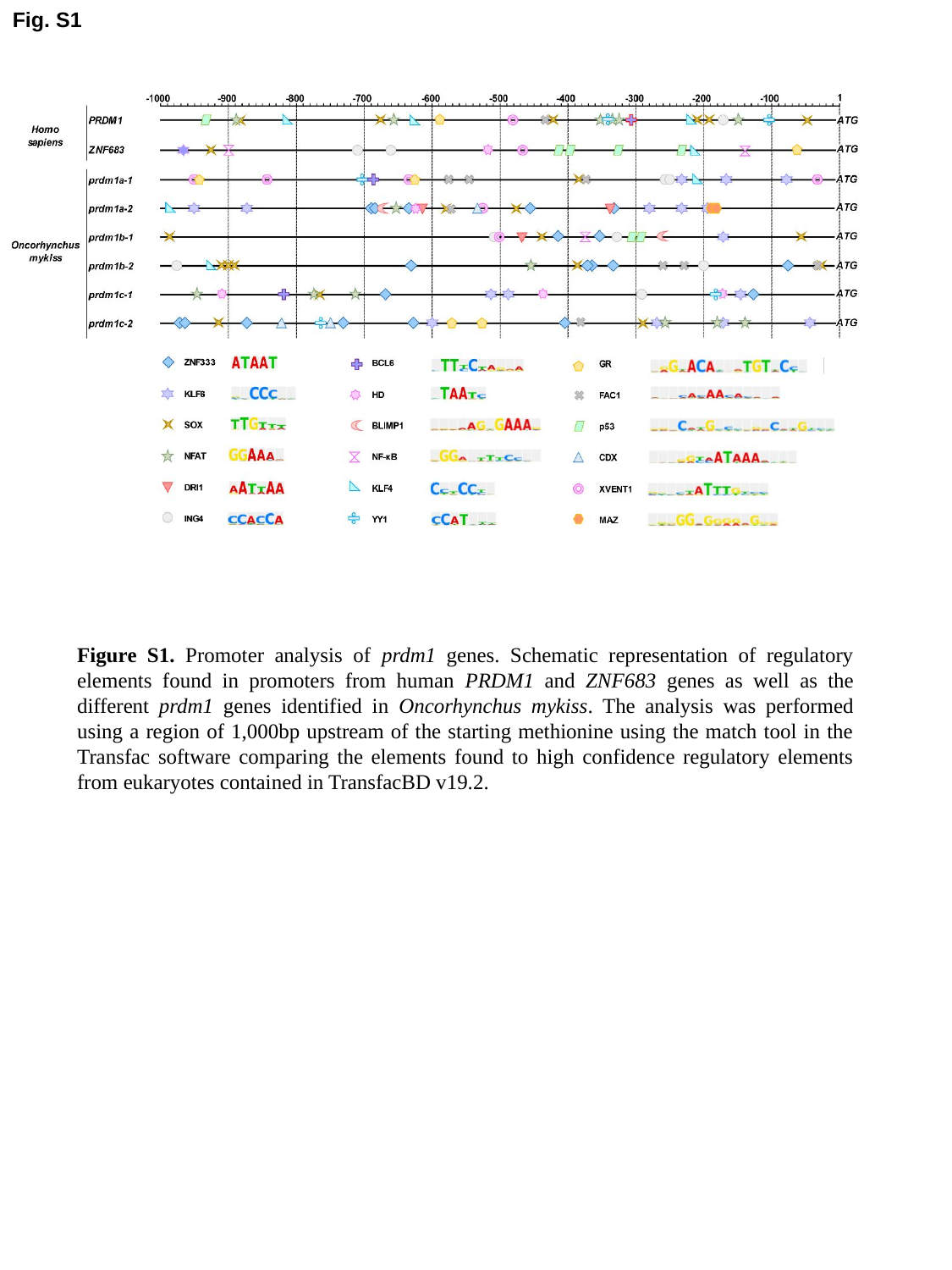

Fig. S1
Figure S1. Promoter analysis of prdm1 genes. Schematic representation of regulatory elements found in promoters from human PRDM1 and ZNF683 genes as well as the different prdm1 genes identified in Oncorhynchus mykiss. The analysis was performed using a region of 1,000bp upstream of the starting methionine using the match tool in the Transfac software comparing the elements found to high confidence regulatory elements from eukaryotes contained in TransfacBD v19.2.

## Slide 2
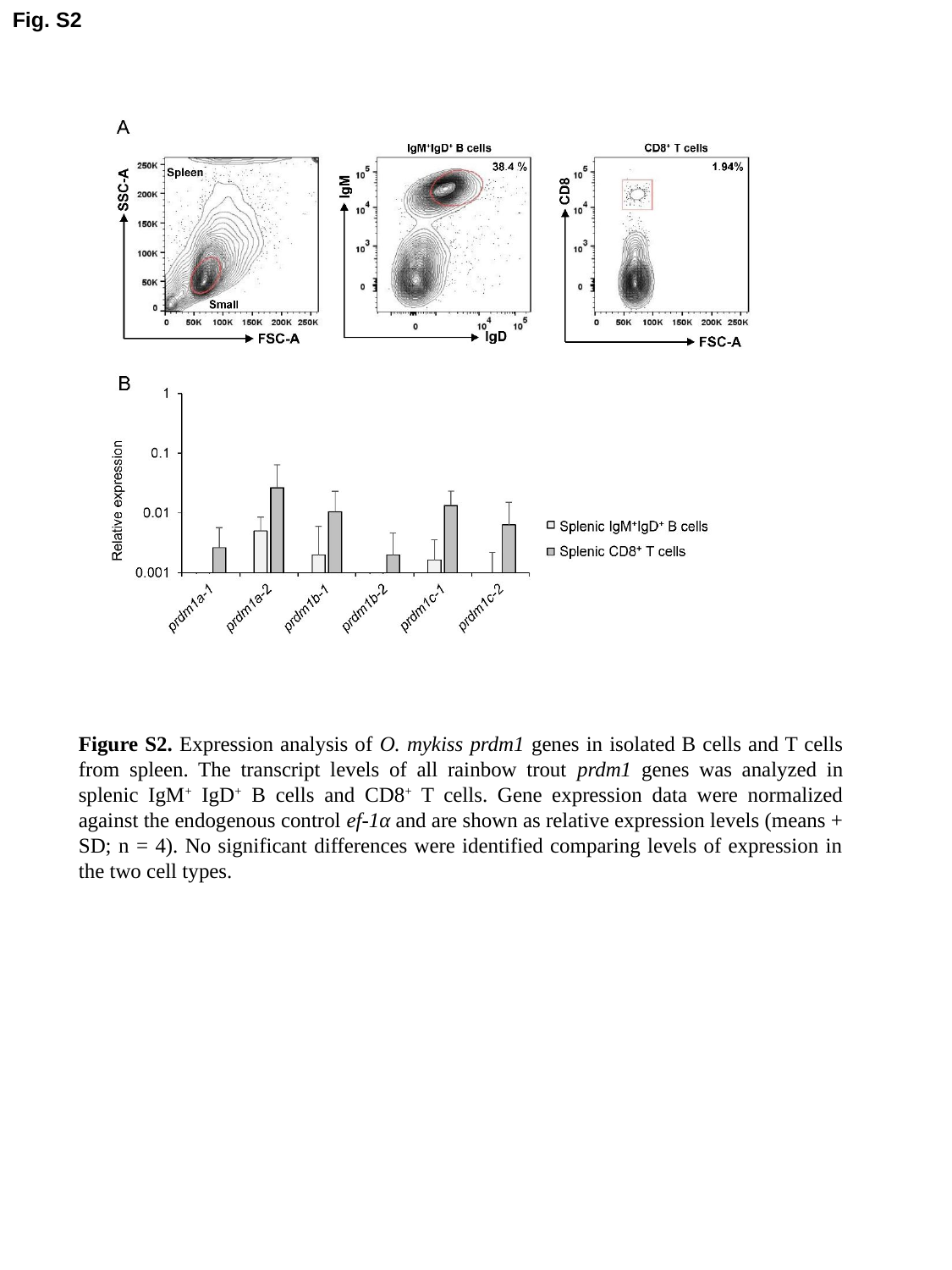

Fig. S2
Figure S2. Expression analysis of O. mykiss prdm1 genes in isolated B cells and T cells from spleen. The transcript levels of all rainbow trout prdm1 genes was analyzed in splenic IgM+ IgD+ B cells and CD8+ T cells. Gene expression data were normalized against the endogenous control ef-1α and are shown as relative expression levels (means + SD; n = 4). No significant differences were identified comparing levels of expression in the two cell types.
